# Glycemic control monitoring in patients with tuberculosis and diabetes: a descriptive study from programmatic setting in Tamil Nadu, India

**DOI:** 10.12688/f1000research.20781.2

**Published:** 2020-06-11

**Authors:** J. Gurukartick, Lakshmi Murali, Hemant Deepak Shewade, Anil G. Jacob, M. M. Samy, D. Dheenadayal, O. P. Aslesh, Ganesh Marimuthu, Ramya Ananthakrishnan, Nalini Krishnan

**Affiliations:** 1 Resource Group for Education and Advocacy for Community Health (REACH), Chennai, India; 2 Government Mohan Kumaramangalam Medical College, Salem, India; 3 State TB cell, Department of Health and Family Welfare,, Chennai, India; 4 International Union Against Tuberculosis and Lung Disease (The Union), Paris, France; 5 The Union South-East Asia Office, New Delhi, India; 6 Government Medical College, Thrissur, India

**Keywords:** tuberculosis treatment, diabetes mellitus treatment, blood glucose, treatment outcome, glycemic control, SORT IT

## Abstract

**Background: **India’s national tuberculosis (TB) programme recommends that among patients with diabetes mellitus and TB, fasting blood glucose (FBG) be recorded at baseline, the end of intensive phase and the end of continuation phase of TB treatment. We conducted this operational research in select districts of Tamil Nadu, India, in 2016 to determine the availability of blood glucose records and glycemic control status during TB treatment.

**Methods: **This was a descriptive study involving secondary programme data. Glycemic control during TB treatment was ‘optimal’ if both baseline and end of intensive phase FBG (during TB treatment) were <130 mg/dl. In the absence of FBG, we used random blood glucose (RBG), with <180 mg/dl as the cut off.

**Results: **Of 438 patients, FBG at baseline, the end of intensive phase and the end of continuation phase were each available in <20%. Glycemic control status was known for 94% (412/438) patients at baseline and for 91% (400/438) during TB treatment. Among those with known glycemic status, glycemic control was not optimal in 77% of patients (316/412) at baseline and in 84% (337/400) during TB treatment. The proportion of patients with unfavourable TB treatment outcomes at the end of intensive phase was 11% (46/438) and at the end of continuation phase was 5% (21/438). We decided against assessing factors associated with glycemic control during TB treatment and association between glycemic control and TB treatment outcomes because glycemic control assessment, if any, was based mostly on RBG values.

**Conclusion: **Among patients with diabetes and tuberculosis, recording of FBG during tuberculosis treatment requires urgent attention.

## Introduction

Worldwide, tuberculosis (TB) remains a major public health problem in low- and middle-income countries. Diabetes mellitus (DM) affected 425 million people in 2017 and is projected to increase to 629 million by 2045 ^1^. Globally, TB and DM are among the top ten causes of death ^2^. Annually, of the estimated 10 million new people with active TB, one million have DM (TB-DM) and this double burden deserves attention ^2– 4^. Considering the bi-directionality of association between TB and DM ^5^, in 2010, a collaborative framework for care and control of TB-DM was developed by World Health Organization (WHO) and the International Union Against Tuberculosis and Lung Disease (The Union) ^6^.

Among patients with TB, DM increases risk of death, recurrence and ‘treatment failure and death’ combined when compared to not living with DM ^7^. DM may result in early mortality and adverse outcomes among patients undergoing TB treatment ^8^.

India has more than 25% of the global TB burden and has the second highest number of people with diabetes after China ^4^. The prevalence of DM among adults with TB was 44% in Kerala state and 25% in Tamil Nadu state ^9^. A recently published study from a district in Tamil Nadu (2014–15) documented a prevalence of 14% ^10^. In 2012, a feasibility study was conducted in India’s revised national TB control programme (RNTCP) settings for bidirectional screening for TB-DM ^11^, based on which in the same year a policy decision was taken to screen all TB patients for DM. The programme also recommends recording of fasting blood glucose (FBG) values at baseline, the end of intensive phase (IP) and the end of continuation phase (CP) of TB treatment ^12, 13^.

Considering that systematic monitoring and recording of glycemic status among patients with TB-DM has been initiated in some districts of Tamil Nadu, this presents a unique opportunity for understanding the same in programme setting. Recording of FBG at baseline and during TB treatment will provide reliable information not only for management of patient’s glycemic status but also to assess the effect of optimal glycemic control on TB treatment outcomes among patients with TB-DM, the evidence for which is limited ^14^.

Therefore, this study was conducted among patients with TB-DM registered for TB treatment in 2016 under the programme in select districts of Tamil Nadu, India. The specific objectives were to determine i) the availability of FBG at various phases of TB treatment as per programme recommendations; and ii) the number (proportion) without optimal glycemic control at baseline and during TB treatment.

## Methods

### Study design

This was a descriptive study involving record review of routinely collected programme data.

### Study setting

**Figure 1.**
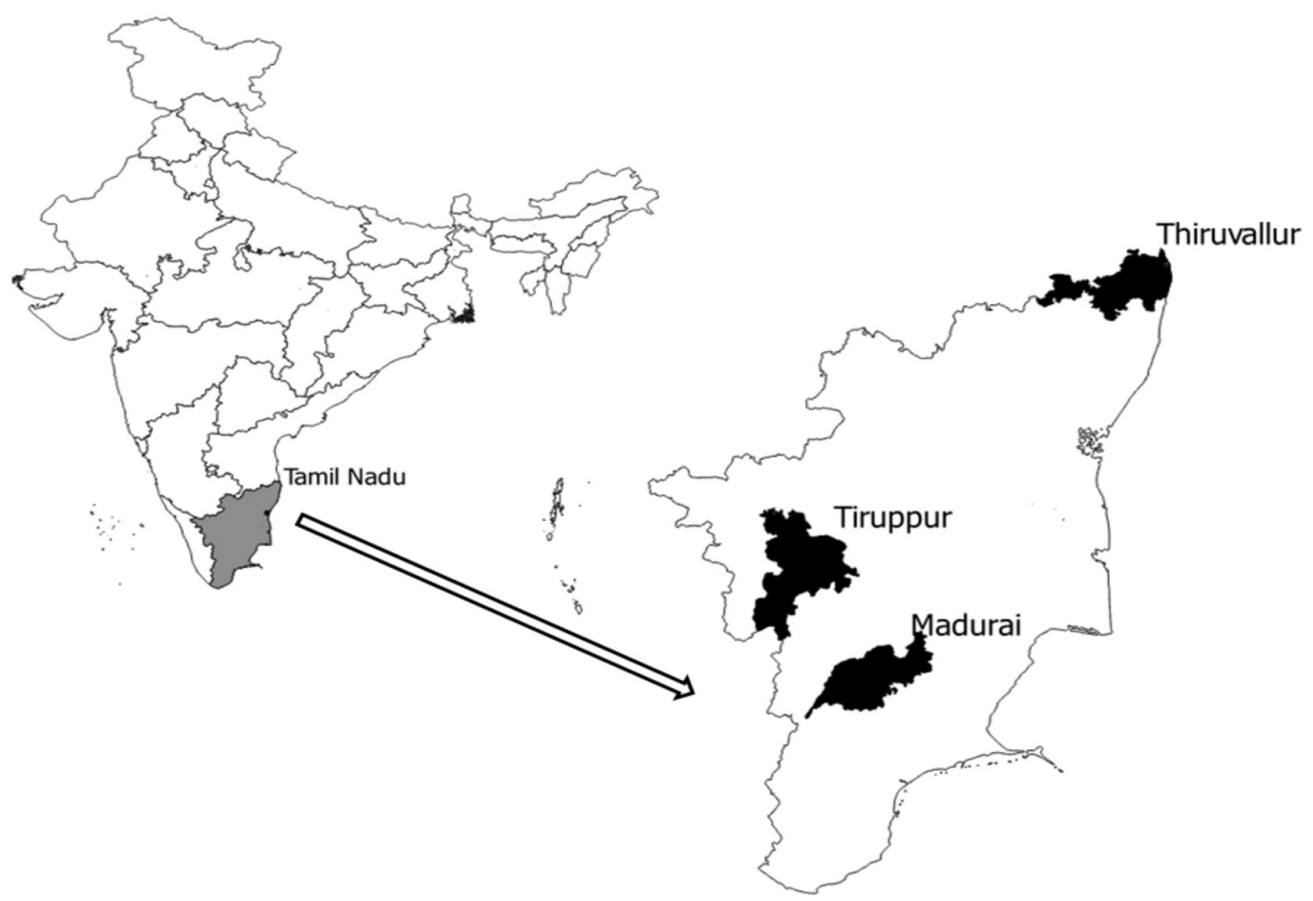
Map of India showing the three study districts in Tamil Nadu, India.

***General setting.*** Tamil Nadu is a state in south India along the eastern coast with predominantly plain terrain. ( Figure 1) Tamil Nadu has a population of 72 million and is made up of 32 administrative districts. The literacy rate is 80% ^15^.

***Study districts.*** The study was conducted in programme setting in Tiruvallur, Madurai and Tiruppur districts of Tamil Nadu. Tiruvallur is a suburb of Chennai (the capital of Tamil Nadu). Tiruppur and Madurai districts are located in west and south Tamil Nadu, respectively ( Figure 1).

Under RNTCP in 2016, each district had one district TB centre, sub-district administrative units called as TB units (Tiruvallur-14, Madurai-21, Tiruppur-16) and designated microscopy centers (Tiruvallur-31, Madurai-35, Tiruppur-20). Diagnosed patients were registered for treatment and entered into the treatment register at the TB unit. Patients received domiciliary directly observed treatment (thrice weekly) which was according to the then national treatment guidelines. TB treatment outcomes (end IP and end treatment outcomes) were recorded in line with WHO recommendations and have been summarized in Box 1 ^16^.

**Box 1. Operational definition for the TB treatment outcomes, revised national TB control programme, India (2016) ^16^.**

**End of TB treatment** – minimum 6 month follow up and maximum up to 31 May 2017

- death, treatment failed, loss to follow up, not evaluated (unfavourable treatment outcome)
- treatment completed, cure (favourable treatment outcome)

*Cured:* ’Cured’ defined as “A pulmonary TB patient with bacteriologically-confirmed TB at the beginning of treatment who was smear- or culture-negative in last month of treatment and on at least one previous occasion”;

*Treatment completion:* ’Treatment completed’ defined as “A TB patient who completed treatment without evidence of failure, but with no record to show that sputum smear or culture results in the last month of treatment and on at least one previous occasion were negative, either because tests were not done or because results are unavailable”;

*Loss to follow-up*: ’Loss to follow-up’ defined as “A TB patient who did not start treatment or whose treatment was interrupted for two consecutive months or more”;

*Treatment failed*: ’Treatment failed’ defined as “A TB patient whose sputum smear or culture is positive at month five or later during treatment”;

*Died*: ’Died’ defined as “A TB patient who dies for any reason before starting or during the course of treatment”;

*Not evaluated:* ’Not evaluated’ defined as “A TB patient for whom no treatment outcome is assigned. This includes cases “transferred out” to another treatment unit as well as cases for whom the treatment outcome is unknown to the reporting unit”.

**End of Intensive phase**

- Death. Loss to follow up, extension of IP and non-conversion at 3 / 4 months (Unfavourable)
- Microbiological conversion (Favourable)

*Sputum conversion*: those who are sputum negative latest by 3 months for new patients and latest by 4 months for retreatment patients

*Sputum non-conversion:* those who are sputum positive at 3 months for new patients and at 4 months for retreatment patients

***TB-DM collaboration.*** One of the activities under TB-DM collaborative framework consists of screening for DM among all patients with TB (initiated in study districts in 2015) and monitoring glycemic control during TB treatment among all patients with TB-DM (initiated in 2016). Patients with TB are first screened with random blood glucose (RBG) at designated microscopy centers; those with RBG≥140 mg/dl (7.8 mmol/l) are assessed using FBG. Those with FBG≥126 mg/dl (7 mmol/l) are referred to higher centers for clinical confirmation of DM and initiation of treatment. This is followed by treatment continuation at the peripheral health centers ^12^.

Patients with TB-DM undergo FBG at the end of IP and at the end of CP and the same are entered in the TB treatment card and register ^12^. There is no specific modification in either DM or TB treatment because of the co-morbidity. TB and DM management is done free of cost.

### Study population

The study population included all patients with TB-DM comorbidity (age ≥15 years) registered under RNTCP between January and September 2016 in Tiruvallur, between January and June 2016 in Madurai and between April and June 2016 in Tiruppur. Patients with multi-drug-resistant TB were excluded from the study.

### Variables, sources of data and data collection

A review of records was conducted between October 2016 and May 2017. Data were collected from TB treatment registers and patient treatment cards using a paper-based data collection form. Each patient was given a unique identifier derived from district code, tuberculosis unit name and TB registration number.

Baseline socio-demographic characteristics, baseline clinical characteristics and treatment outcomes (end IP and end treatment) were collected. In addition, blood glucose values at baseline, at the end of IP and at the end of CP along with the type of test (FBG or RBG) were collected.

### Analysis and statistics

Data collected were double-entered, validated and analysed using EpiData (version 3.1 for entry and version 2.2.2.183 for analysis; EpiData Association, Odense, Denmark). Frequency and proportions were used to summarize the key analytic outputs.

The cut off for optimal glycemic control was <130 mg/dl (7.2 mmol/l) for FBG and <180 mg/dl (10 mmol/l) for RBG. The cut off (to define optimal control) for post-prandial blood glucose was used for RBG ^17^. If both FBG and RBG were known, FBG was used for determining optimal glycemic control. ‘Glycemic control during TB treatment’ was classified as ‘optimal’ (if both baseline and end IP values were optimal), ‘not optimal’ (if baseline and/or end IP were not optimal) or ‘missing’ (if both baseline and end IP values were missing; or if one of the two values were missing and the other was optimal). TB treatment outcomes (both end IP and end treatment) were reclassified as favourable or unfavourable ( Box 1). **

### Ethics approval

Ethical approval was obtained from the Institutional Ethics Committee of the Resource Group for Education and Advocacy for Community Health, Chennai, Tamil Nadu, India, and the Ethics Advisory Group of the International Union Against Tuberculosis and Lung Disease, Paris, France. As the study involved review of existing patient records, a waiver for informed consent was sought and approved by the respective ethics committees. We conducted the study after receiving approval from the State Tuberculosis Officer.

## Results

Of 5115 patients registered with TB, there were 438 (8.6%) patients with TB-DM: 270 from Tiruvallur, 114 from Madurai and 54 from Tiruppur. The baseline socio-demographic and clinical characteristics of patients with TB have been summarized in Table 1. Extracted values for each participant are available as *Underlying data* ^18^.

**Table 1.** Baseline socio-demographic and clinical profile of patients with TB-DM registered in RNTCP in select districts of Tamil Nadu, India (Jan–Sept 2016).

Variable	Categories	N (%)
Total		438 (100)
Age in years	15–44	102 (23)
	45–64	290 (66)
	≥65	46 (11)
Sex	Male	332 (76)
	Female	106 (24)
Occupation	Professional	15 (3)
	Skilled worker	75 (17)
	Unskilled worker	261 (60)
	Unemployed	2 (<1)
	Missing	84 (19)
Place of residence	Urban	164 (37)
	Rural	267 (61)
	Urban slum	7 (2)
TB category	New	373 (85)
	Previously treated	
	Recurrent	39 (9)
	LFU	16 (4)
	Failure	2 (<1)
	Others	8 (2)
Site of TB	Pulmonary	412 (94)
	Extra-pulmonary	26 (6)
Sputum status	3+	66 (15)
	2+	79 (18)
	1+	92 (21)
	Scanty	18 (4)
	Negative	92 (21)
	Missing	3 (<1)
HIV status	Positive	7 (2)
	Negative	423 (97)
	Unknown	8 (2)
Weight in kg	<30	3 (<1)
	30–59	346 (79)
	≥60	89 (20)
Smoking status	Yes	33 (8)
	No	93 (21)
	Missing	312 (71)
Alcoholic status	Yes	49 (11)
	No	76 (17)
	Missing	313 (72)
Hypertension	Yes	19 (4)
	No	105 (24)
	Missing	314 (72)
Treatment initiation delay	No delay	328 (75)
	Delay (>7 days)	110 (25)

TB-DM, tuberculosis and diabetes mellitus; LFU, loss to follow-up; RNTCP, Revised National Tuberculosis Control Programme.

Blood glucose records (either FBG or RBG) were available for 94% at baseline, 79% at the end of IP and 59% at the end of CP. However, FBG at baseline, at the end of IP and at the end of CP were each available for less than 20% patients. RBG at baseline, at the end of IP and at the end of CP were each available for 76%, 60% and 43%, respectively ( Table 2).

**Table 2.** Glycemic control at different stages of treatment among patients with TB-DM registered in RNTCP in select districts of Tamil Nadu, India (Jan–Sept 2016) (n=438).

	Optimal Control * N (%) **	Not optimal control N (%) **	Missing N (%) **
**Based on RBG**			
At baseline	59 (14)	270 (62)	109 (25)
At end intensive phase	98 (22)	168 (38)	172 (39)
At end continuation phase	82 (19)	104 (24)	252 (58)
**Based on FBG**			
At baseline	37 (8)	48 (11)	353 (81)
At end intensive phase	43 (10)	39 (9)	356 (81)
At end continuation phase	51 (12)	22 (5)	365 (83)
**Based on PPBG**			
At baseline	22 (5)	63 (14)	353 (81)
At end intensive phase	27 (6)	53 (12)	358 (82)
At end continuation phase	33 (8)	40 (9)	365 (83)
**Based on RBG or FBG ^^^**			
Baseline	96 (22)	316 (72)	26 (6)
At end intensive phase	141 (32)	207 (47)	90 (21)
At end continuation phase	133 (30)	126 (29)	179 (41)
During TB treatment ^#^	63 (14)	337 (77)	38 (9)

RNTCP, Revised National Tuberculosis Control Programme; RBG, random blood glucose; FBG, fasting blood glucose; PPBG, 2-hour post prandial blood glucose. *RBG, FBG, PPBG cut off of 130, 180 and 180 mg/dl respectively; **row percentage; ^FBG preferred over RBG if available; ^#^‘optimal’ (both baseline and IP status optimal), ‘not optimal’ (any one status was not optimal) and missing (both were missing).

Glycemic control status was known for 94% (412/438) patients at baseline and for 91% (400/438) during TB treatment. Among those with known glycemic status, glycemic control was not optimal in 77% (316/412) patients at baseline and in 84% (337/400) during TB treatment.

****In total, 46 patients (10%) had unfavourable end IP outcomes (5% sputum non-conversion, 2% death and 3% not evaluated); 21 patients (5%) had unfavourable end treatment outcomes (1% treatment failed, 3% deaths and 1% not evaluated).

We decided against assessing factors associated with glycemic control during TB treatment and association between glycemic control and TB treatment outcomes because glycemic control assessment, if any, was based mostly on RBG values, which are unreliable.

## Discussion

This operational research had two key findings. First, though blood glucose values were available during baseline and end IP in four-fifths of patients, most of these values were RBG. FBG was not consistently recorded during baseline, the end of IP and the end of CP (as per programme guidelines). Routinely, patients with TB screened at peripheral health centers using RBG and/or FBG are referred to higher institutions for clinical confirmation. Even if FBG is not measured at peripheral health center at baseline, it ought surely to be measured at the higher center. This FBG at baseline, which is at least expected for all newly diagnosed DM patients at the time of registration for TB treatment, is not reflected in the RNTCP records (only 19% have baseline FBG records). There is one possible reason for this. During data collection, we found that RBG was predominantly measured and recorded as it was the blood glucose test mentioned in the TB treatment card. In a study conducted in 18 randomly selected districts of India, poor documentation of DM status among TB patients from marginalised and vulnerable populations (2016–2017) was observed ^19^.

 Second, a large number of patients did not have optimal glycemic control at baseline and during TB treatment. Though these findings are mostly based on RBG that was available, our figures are comparable with findings from another study by Mahishale *et al*. ^20^ at a private tertiary care facility in Karnataka, India, where poor glycemic control (HbA1C ≥7%) at baseline was seen in 67% (423/630) patients. Another study in India from programme setting by Nandakumar *et al*. ^21^ identified 667 patients with TB-DM, 36% cases had known (minimum three blood glucose values available, at least one month apart) diabetic control status during the treatment, of which 43% cases were under glycemic control. However, the cut offs (FBG <100 mg/dl and RBG <140) used were lower than the ones used by us. The cut-off values used by us were as per international recommendations ^22^.

### Implications for policy and practice

This study has two key policy implications. First and foremost, systematic recording of FBG should be improved. Though HbA1c has been described as the gold standard for glycemic control, as per programme recommendations FBG should be systematically recorded in the treatment cards (considering the lack of access to HbA1c in programme settings) ^12, 13^. One potential solution for this is an incremental administrative change that may easily be incorporated. Newly printed treatment cards replacing RBG with FBG would suffice.

Second, poor glycemic control indicates that there is an urgent need to improve glycemic management among patients with DM. Tamil Nadu has a well-established primary health care system where DM treatment is provided through the peripheral health centres. Existing DM treatment guidelines, if any, should be reviewed to ensure that management is as per international recommendations ^17, 23– 26^. Poor glycemic control has been reported among patients with DM in Asian countries ^27, 28^. Programmatic interventions to maintain a line list of registered patients with DM (in line with TB and HIV programmes) may be considered. This will ensure proper tracking of DM treatment outcomes ^29^. Integrating care of communicable and non-communicable diseases (such as TB and DM, respectively) has also been recommended in literature ^30^.

### Strengths and limitations

To our knowledge, this is the first study which has systematically reported the recording of blood glucose values among patients with TB-DM at various phases of TB treatment in programmatic setting in India. We found two similar studies; however, they did not review glycemic control measurement as per the recent 2016 programme recommendations ^20, 21^. This study was conducted in the programme setting; therefore, findings are reflective of reality on the ground. Double data entry and validation minimized data entry errors.

 There was a major limitation. As RBG was the most frequent type of blood glucose value recorded, glycemic control reported should be interpreted with caution. For the same reason analysis of the association between glycemic control and TB treatment outcome would have been meaningless and hence, was not performed. RBG is a screening test for diabetes and is not recommended for monitoring glycemic control ^22^.

## Conclusions

In this operational research, involving patients with TB-DM in programmatic setting from South India, we found FBG was not consistently recorded as per programme guidelines (at baseline, the end of intensive phase and the end of continuation phase of TB treatment). The glycemic control status (mostly based on random blood glucose instead of fasting blood glucose or HbA1c) was not reliable to perform a meaningful analysis of its effect on TB treatment outcomes. This calls for an urgent review of the TB-DM collaborative services to improve the recording of glycemic control during TB treatment.

## Data availability

### Underlying data

Figshare: Data set for Gurukartick study 2016. https://doi.org/10.6084/m9.figshare.9902180 ^18^.

File ‘Dataset_v2’ contains all de-identified variables extracted for this study, alongside a codebook explaining all abbreviations and values.

Data are available under the terms of the Creative Commons Zero “No rights reserved” data waiver (CC0 1.0 Public domain dedication).
