# Supplementary material for: Glycemic control monitoring in patients with tuberculosis and diabetes: a descriptive study from programmatic setting in Tamil Nadu, India
Source: F1000Res. 2020 Jun 11;8:1725. Originally published 2019 Oct 7. [Version 2] doi: 10.12688/f1000research.20781.2 (PMC7238456; doi:10.12688/f1000research.20781.2)
Supplement: Peer review history

# Peer review: Reviewer response for version 1

**DOI:** 10.5256/f1000research.22856.r62134

**Published:** 2020-05-19

**Authors:** Aniwada Elias Chikee

**Affiliations:** 1 Department of Community Medicine, College of Medicine, University of Nigeria Teaching Hospital, Enugu, Nigeria

The work is good and well-articulated. The authors could not found previous work that hampered testing for association as well as discussion. This is genuine and tenable.

 If possible, the authors to replicate the study using longitudinal or prospective challenge in another study to overcome the limitation of RBG against FBG. This will help in appreciation of difficult associated with TB-DM co-morbidity both for patients and TB control programme

Is the work clearly and accurately presented and does it cite the current literature?

Yes

If applicable, is the statistical analysis and its interpretation appropriate?

Yes

Are all the source data underlying the results available to ensure full reproducibility?

Yes

Is the study design appropriate and is the work technically sound?

Yes

Are the conclusions drawn adequately supported by the results?

Yes

Are sufficient details of methods and analysis provided to allow replication by others?

Yes

Reviewer Expertise:

NA

I confirm that I have read this submission and believe that I have an appropriate level of expertise to confirm that it is of an acceptable scientific standard.

# Response

**Published:** 2020-05-20

**Authors:** Hemant Deepak Shewade

**Affiliations:** International Union Against Tuberculosis and Lung Disease, India

Dear Aniwada, Thank you for approval and constructive comments. Your suggestions for a longitudinal study in the future are well taken. With regards, Hemant

# Peer review: Reviewer response for version 1

**DOI:** 10.5256/f1000research.22856.r57043

**Published:** 2019-12-09

**Authors:** Madhavi Bhargava

**Affiliations:** 1 Centre for Nutrition Studies, Yenepoya University, Mangalore, Karnataka, India

It is a well written manuscript. Few suggestions: 

1. For the 3 ^rd^ paragraph of the introduction, reference 9: The looming co-epidemic document ( https://www.theunion.org/what-we-do/publications/technical/low-resolution/25383_TCB_Report_LR.pdf) mentions figures from TN from a 2012 study (see page 10 of this document). There is one study from Tamil Nadu in which diabetes was documented in 163 (14%) of 1131 TB patients (10.4103/ijcm.IJCM_179_16). Consider using this for more recent, same state figures.
2. Box 1 seems redundant as the authors decide against analyzing the outcomes based on RBGs. The information available in the results section, fourth paragraph is adequate and self-explanatory.
3. Since this is a record review, it will be good to know the denominator of all TB patients diagnosed in all these three tuberculosis units of which, the authors found 438 patients with TB-DM. This will add important information for the program, as well as future studies.

Is the work clearly and accurately presented and does it cite the current literature?

Yes

If applicable, is the statistical analysis and its interpretation appropriate?

Yes

Are all the source data underlying the results available to ensure full reproducibility?

Yes

Is the study design appropriate and is the work technically sound?

Yes

Are the conclusions drawn adequately supported by the results?

Yes

Are sufficient details of methods and analysis provided to allow replication by others?

Yes

Reviewer Expertise:

NA

I confirm that I have read this submission and believe that I have an appropriate level of expertise to confirm that it is of an acceptable scientific standard.

# Response

**Published:** 2020-06-08

**Authors:** Hemant Deepak Shewade

**Affiliations:** International Union Against Tuberculosis and Lung Disease, India

REVIEWER: Madhavi Bhargava

 REVIEWER

 For the 3rd paragraph of the introduction, reference 9: The looming co-epidemic document (https://www.theunion.org/what-we-do/publications/technical/low-resolution/25383_TCB_Report_LR.pdf) mentions figures from TN from a 2012 study (see page 10 of this document). There is one study from Tamil Nadu in which diabetes was documented in 163 (14%) of 1131 TB patients (10.4103/ijcm.IJCM_179_16). Consider using this for more recent, same state figures.

  AUTHOR RESPONSE

 Thank you for the comment. We have included this reference in the third paragraph of the introduction.

 REVIEWER

 Box 1 seems redundant as the authors decide against analyzing the outcomes based on RBGs. The information available in the results section, fourth paragraph is adequate and self-explanatory.

 AUTHOR RESPONSE

 Thank you for the comment. We agree that we have not analysed the outcomes based on glycemic control. However, as we have presented the overall treatment outcomes in the results section, we would request that the box 1 (operational definition of treatment outcomes) be retained. We hope this is fine.

 REVIEWER

 Since this is a record review, it will be good to know the denominator of all TB patients diagnosed in all these three tuberculosis units of which, the authors found 438 patients with TB-DM. This will add important information for the program, as well as future studies.

 AUTHOR RESPONSE

 Thank you for the comment. We have included this information (438 TB-DM out of 5115 TB patients. Please see first line of results narrative.
